# Sex-specific efficacy of deslorelin in downregulating reproductive activity in the grey mouse lemur (*Microcebus murinus*)

**DOI:** 10.1590/1984-3143-AR2023-0137

**Published:** 2024-11-18

**Authors:** Aude Noiret, Fabienne Aujard, Jeremy Terrien

**Affiliations:** 1 Unité Mécanismes Adaptatifs et Evolution (MECADEV), UMR 7179, CNRS, Muséum national d’Histoire naturelle, Brunoy, France

**Keywords:** seasonal reproduction, deslorelin, grey mouse lemur, sex-specific physiology

## Abstract

Deslorelin is a GnRH agonist used in veterinary medicine to temporarily inhibit reproduction in domestic animals and is sometimes tested in captive species in zoo to control population or tame aggressive behaviours in males. However, some studies have revealed the inefficacy of deslorelin specifically in males, contrary to females that follow a classic long-term inhibition of the reproductive hypothalamic-pituitary axis through sexual steroid negative feedback. We implanted 5 males and 6 females grey mouse lemurs (*Microcebus murinus*), long-day breeders that display a complete inhibition of the reproductive system during winter, at the end of the short-day period, a few weeks before the breeding season. Contrary to females, which exhibited a classic inhibitory response to deslorelin, males’ testosterone levels increased as well as their testis size, which suggests a sex-specific sensitivity to the negative feedback of sexual steroids before the mating period. We propose that this sex-imbalance is related to the different life-history of males as opposed to females concerning reproductive tasks and behaviour.

## Introduction

Seasonal breeders are known for their sex-specific reproductive axis regulations (Ball and Ketterson, 2008). In many mammal species, males show early photorefractoriness to short day (SD) exposure, while females maintain an endogenous cycle which synchronizes with long day (LD) transition (Perret and Aujard, 2001; Prendergast, 2005). These features likely support males and females’ specific agendas regarding reproduction. Indeed, while males lose sensitivity to short photoperiod in late winter, they prepare spermatogenesis in anticipation of the mating season and engage in territory competition and courtship (Key and Ross, 1999; Lane et al., 2010). In contrast, females stay sexually quiescent until the photo-transition to summer as they rely on a reserve of gametes from birth and delay reproductive activity until mating, gestation, lactation and young care, that occur a few weeks later (Czenze et al., 2017). Hence, there is a sex difference in photosensitivity (loss in males, maintenance in females) in late winter to control the sexual activity (i.e. either reactivation or quiescence in males and females, respectively). Such time-shift in sexual reactivation may reveal a difference between males and females’ sensitivity to the sexual hormones inhibitory action on the reproductive axis during late winter. Whether the neuroendocrine regulation of the reproductive axis differs between males and females during this critical period remains to be demonstrated.

To tackle this question, we investigated sex-specific responsiveness to sexual hormone negative feedback before the mating period by implanting grey mouse lemurs (*Microcebus murinus*) with a GnRH agonist, deslorelin (Padula, 2005). As an agonist of GnRH, deslorelin primarily stimulates the release of sexual hormones in both males and females, which later translates into long-term inhibition of the hypothalamus-pituitary axis through a negative feedback (Trigg et al., 2006), resulting in a chemical sterilization. However, in some species, while deslorelin is efficient on females (Fontaine, 2015; Goericke-Pesch and Wehrend, 2012; Silvestre et al., 2009), males respond poorly (Aspden et al., 1998; Eymann et al., 2007; Lincoln, 1987) with no modification of sexual activity. While the results in these species did not meet the expectations for an application in veterinary medicine (population regulation, inhibition of aggressive behaviour), the differences between males and females in the responsiveness to the molecule represents a good opportunity to discuss sex-specific reproductive regulation in a fundamental and evolutionary point of view.

As seasonal breeders, female *Microcebus murinus* show endogenous oestrous cycles that are synchronized with the LD photoperiodic exposure, while males enter in an early photorefractoriness after 14 weeks of SD exposure, which translates into rising testosterone levels and testis recrudescence (Perret and Aujard, 2001; Terrien et al., 2017). While these seasonal regulations are naturally expressed under natural conditions in Madagascar, they are maintained in captivity under an artificial lightening. Indeed, the alternation between 6 months of short photoperiod (10 hours of light per day) and 6 months of long photoperiod (14 hours of light per day) mimics the seasonal photoperiodic changes between winter and summer, respectively, in Madagascar). Although animals are housed in constant conditions of high temperature (25°C) and access to food for more than 50 years, the seasonal features of this species are maintained in captive conditions at both the behavioural and physiological levels (Perret and Aujard, 2001; Terrien et al., 2017). In a pilot study, a male was used to check on deslorelin efficacy and showed very low urinary testosterone concentration (7.8 ng.mg Creat.^-1^) compared to two non-implanted males (27.6 and 60.1 ng.mg Creat.^-1^) 15 weeks after implantation, despite large testis size. Here, we intended to test the efficacy of deslorelin in mouse lemurs, neither for contraceptive implications in a captive setting, nor for understanding broader wildlife management implications, but rather as a fundamental test to assess its efficiency on the inhibitory action on the reproductive axis in a seasonal species. For this, we implanted both sexes with deslorelin before males and females’ sexual reactivation, i.e. before the second half of winter to test for a sex difference in response to the increase in sexual hormones (testosterone and oestrogen). Despite originally expecting an inhibition of sexual activity in both sexes, we show instead contrasting results between males and females, and question this outcome in relation to the sex-specific life history of the species.

## Methods

### Animals and ethical concerns

Thirty grey mouse lemurs (*Microcebus murinus*), 14 males and 16 females all aged from 2 to 4 years and raised in good health in the breeding colony of Brunoy (MNHN, France, license approval n◦ E91-114-1), were included in the experiment. Temperature and humidity were maintained constant (24–26°C and 55%, respectively). The lemurs were fed with a fresh home-made mixture (egg, concentrate milk, cereals, spicy bread, cream cheese, and water) complemented with fresh banana, and were provided with *ad libitum* water. All described experimental procedures were approved by the Animal Welfare board of the UMR 7179, the Cuvier Ethics Committee for the Care and Use of Experimental Animals of the Muséum national d’Histoire naturelle, authorized by the Ministère de l’Enseignement Supérieur, de la Recherche et de l’Innovation (n◦14075-2018031509218574) and complied with the European ethic regulations for the use of animals in biomedical research. Animals were all single-housed and kept in general housing conditions during the whole experiment.

### Manipulation of the reproductive axis

Eleven animals (DIM deslorelin-implanted males, N=5; or DIF, deslorelin-implanted females, N=6) received a deslorelin implant (Suprelorin 9.4 mg) 6 weeks after short-day transition (SD: 10 hours light/14 hours dark), during early winter, at the time when both sexes reached an inactive sexual state (Perret and Aujard, 2001). Implants were placed on the interscapular zone under general anaesthesia (Alfaxalone 20 mg/kg IM + lidocaine injection at the implantation site). The implants were well supported, no itching lesions were observed and the wounds closed by themselves 2 days after the procedure. An additional surgical procedure was performed under general anaesthesia 50 to 35 days before LD transition for males and 1 month after transition to summer for females, in order to remove as much as implant as possible. Although such removal is not usually intended for veterinary practices, it allowed to verify the delay needed for the recovery of normal reproductive activity. At removal time, the implants were found dissociated in several pieces. Although, the removal was complete and successful in most cases, some small implant pieces were left in the animals in some cases. In parallel, non-implanted animals (NIM non-implanted males, N=9; or NIF, non-implanted females, N=10) were also monitored.

### Assessment of reproductive activity

**Figure 1:**
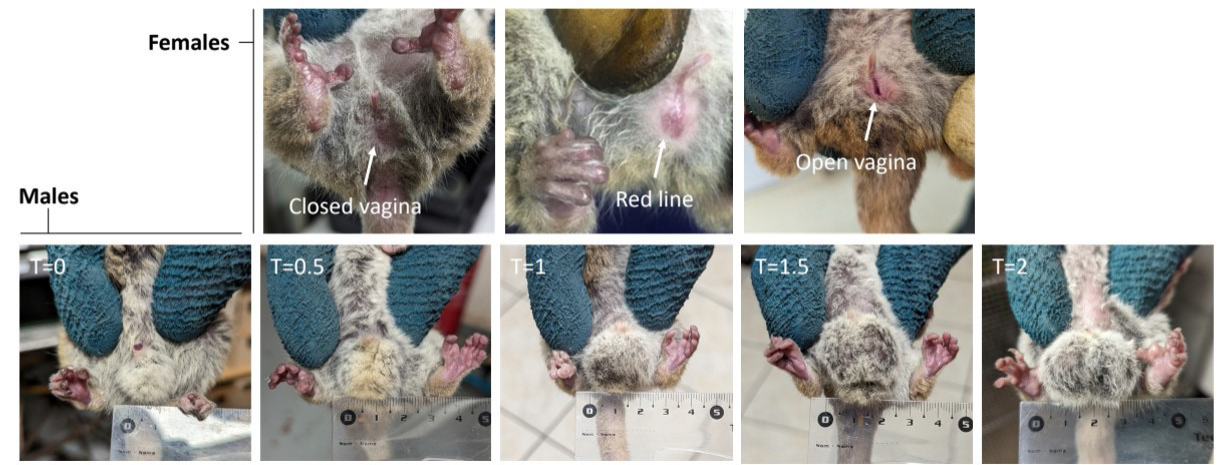
Photographs of the reproductive state in female (upper panels) and male (lower panels) mouse lemurs. Females reproductive status changes from inactive (closed vagina; left upper panel) to the formation of a red line (middle upper panel) and the production of an oestrus (open vagina; right upper panel). In males, testis score ranges from 0 (inactive) to 2 (active), according to size and consistency (left to right lower panels).

Sexual status was monitored during 20 weeks from implantation until transition to long-days (summer season). Females were checked by visual examination to determine whether they entered either proestrus or oestrus manifestation, which is easily observable in this species (Figure 1). In males, testis size (TS) was monitored each month (rated from 0 to 2 based on their size and consistency: 0 = testes are up in the abdominal cavity and scrotal sacs are loose; 0.5 = testes descend and measure 1 cm put together; 1 = 2 cm; 1.5 = 3cm; 2 = 4cm) (Figure 1). Sexual hormone levels (urinary 17-beta-Estradiol in females, plasma and urinary testosterone in males) were assayed by ELISA after 2 months of implantation in all animals (“17-beta-Estradiol” in pg.ml^−1^, IBL, ref RE52041; “Testosterone” in ng.ml^−1^, Abcam, ref ab108666). Creatinine concentration (mg.ml^−1^) was used to normalize all urine measurements as an indicator of renal filtration activity (MicrovueTM Creatinine Elisa kit, Quidel R Corporation, ref 8009). Results are thus expressed in ng.mg Creat.^−1^ or pg.mg Creat.^−1^ for urinary testosterone and 17-beta-Estradiol, respectively.

## Results

### Effect of deslorelin implant in female mouse lemurs

All deslorelin-implanted females (DIF, N=6) showed early manifestation of reproductive axis activity between day 7 to 13 after implantation, i.e. 7 to 8 weeks after winter onset, as three females were in proestrus and 2 showed true open vaginas (Figure 1B). Females were checked 2 weeks after, and one showed a cicatricial line at the vaginal region, indicative of an oestrus that occurred a few days before. All females then entered into an inactive reproductive state (no oestrus) for the rest of winter, until photo-transition to LD. After LD transition, DIF did not show sexual reactivation, while estruses were observed in non-implanted females (NIF) about two weeks after LD transition (17 ± 3 days).

**Figure 2:**
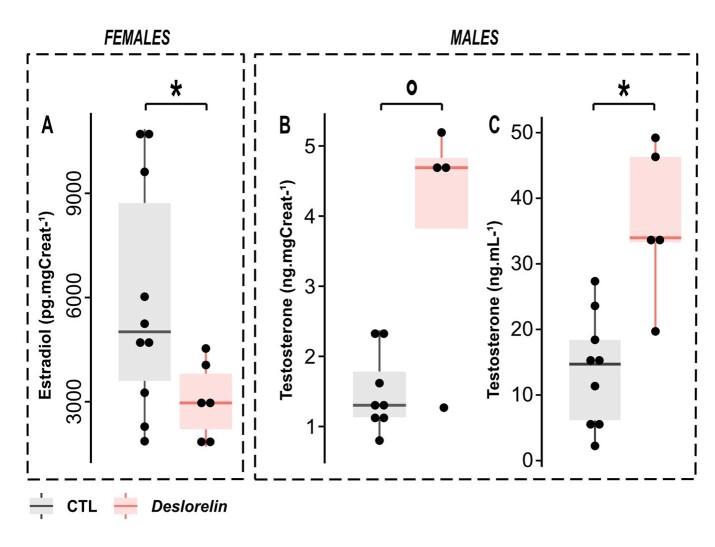
Sexual hormone levels in females (Estradiol) and males (Testosterone), in CTL animals (in grey) or after 2 months of deslorelin implantation (in red); (A) Estradiol concentration in urine in females corrected by creatinine concentration (ng.mgCreat^-1^); (B) Testosterone concentration in urine in males corrected by creatinine concentration (ng.mgCreat^-1^); (C) Testosterone concentration in plasma in males (ng.mL^-1^). °: p-value < 0.1; *: p-value < 0.05.

The visual observations of sexual inhibition during winter in DIF were consistent with oestrogen concentrations in urine, which were significantly lower in DIF than in NIF (3034 ± 1112 pg.mgCreat^-1^ in DIF vs. 5910 ± 3328 pg.mgCreat^-1^ in NIF; W=11, p-value= 0.042 Figure 2A) 2 months after implantation (during the second half of winter).

### Effect of deslorelin implant in male mouse lemurs

In males, testis recrudescence began at week 9 after the beginning of the SD period, one month after deslorelin implantation (DIM: testis size was > 0.5 by 10 ± 1.2 weeks after SD transition), while it started around week 14 for non-implanted males (NIM: testis size was > 0.5 by 14 ± 2.2 weeks after SD transition). This earlier recrudescence in DIM was accompanied by a tendency for higher testosterone concentrations in urine until week 21 after SD transition (3.9 ± 1.8 ng.mgCreat^-1^ in DIM vs 1.5 ± 0.6 ng.mgCreat^-1^ in NIM; W=27, p-value= 0.073, Figure 2B). Plasma analysis significatively confirmed the augmentation of testosterone levels in deslorelin-implanted males compared to non-implanted animals (36.5 ± 11.8 ng.mL^-1^ in DIM vs 13.8 ± 8.5 ng.mL^-1^ in NIM; W= 43, p-value= 0.004, Figure 2C).

## Discussion

As seasonal breeders, grey mouse lemurs reproduce during a relatively short time-frame during long days. To prepare for the mating event, males show earlier gonadal activity as compared to females, preparing sperm stocks before the phototransition to long days. Moreover, this primate species present specific reproductive life history traits (sperm competition, territoriality, exclusive maternal care), which contribute to the male-female gap in the timing and energy allocated to biological and behavioural tasks regarding reproduction and young care. Hence, grey mouse lemurs are good candidates to study sex-specific central regulation of the reproductive axis and sensitivity to the sexual steroids negative feedback, using a GnRH agonist, deslorelin.

### The inhibitory effect of deslorelin is sex-specific in wintering mouse lemurs

In wintering quiescent female mouse lemurs, the pattern of successive reactivation followed by inhibition of reproductive activity after deslorelin implantation is very similar to what is observed -and targeted- in domestic mammals such as cats, dogs, ferrets or dairy cows (Fontaine, 2015; Goericke-Pesch and Wehrend, 2012; Silvestre et al., 2009; Walter et al., 2011). Indeed, deslorelin implantation in these species triggers an initial upregulation of the reproductive axis, followed by a subsequent desensitization leading to a quiescent reproductive state. Given the effects observed in the pilot study in one single male (lowering of urinary testosterone despite large testes), we initially thought that the early recrudescence of testis size following deslorelin implantation was not synonym of testicular activity. Testis could be “frozen” in a big shape after an initial reactivation, showing no hormonal production, nor exocrine function, which ultimately meant that males developed desensitization of their gonadotropic cells, as it is described in dogs (Junaidi et al., 2003), and as we observed in female mouse lemurs. In our large-scaled manipulation however, we observed an opposite effect in male mouse lemurs, with an increase in testis size associated with the increase in urinary testosterone levels, suggesting the inefficiency of the negative feedback loop. Due to limited possibilities in blood sampling volume in those small animals, we did not assay for LH or FSH levels to acknowledge true desensitization of the gonadotropic axis, yet it is the general understanding about the action mechanism of GnRH agonists. In many species, males showed significant reduction of FSH and testosterone levels after deslorelin implantation, such as in the plasma of dogs (Junaidi et al., 2003), cheetas, cats and ferrets (Bertschinger et al., 2006; Fontaine, 2015; Goericke-Pesch and Wehrend, 2012), as well as in the stools of eulemurs (Ferrie et al., 2011) and baboons (Young, 2013). Sex-specific effects of deslorelin exposure were previously described in the common brushtail possum, where females responded by a disruption of the normal estrous-cycle after an acute increase of LH, while males remained fertile after chronic deslorelin exposure and even sired as many offspring as the CTL males (Eymann et al., 2007). However, plasma testosterone and FSH levels decreased in response to deslorelin in possums, which is yet another contradiction with our results. The male possums also lost the ability to respond to a surge of GnRH by an increased LH peak. Males of different species showed poor response to chronic GnRH agonist treatment, such as the marmoset monkey (Lunn et al., 1992; Lunn et al., 1990), red deer stag (Lincoln, 1987), tammar wallaby (Herbert et al., 2004) and bulls (D'Occhio et al., 2000). In bulls, LH release was associated with increased secretion of testosterone, persistent for the duration of deslorelin treatment (Aspden et al., 1998). It was suggested that bulls treated with GnRH agonist undergo the classical desensitisation of the pituitary and downregulation of endocrine function, but that other testicular factors could be involved in maintaining LH secretion, such as an increase in the rate of transcription and translation of LH β-subunit mRNA to LH (Aspden et al., 1998). Increased testosterone secretion in deslorelin-treated bulls was also associated with increased levels of testicular steroidogenic acute regulatory (StAR) protein and steroidogenic enzymes (Aspden et al., 1998). Whether this pattern is also implicated in the regulation of spermatogenesis in male mouse lemurs remains to be explored.

### Sex-specific response to deslorelin as a result of different reproductive constraints

From the literature, it appears that deslorelin inefficacy is only observed in males, although the test is often made in the one sex - except for possums and marmosets. During the period of sexual activity, the feedback control of sexual steroids seems to emerge for different reasons in both sexes. In females, it regulates the estrous cycle, controlling the LH pulse and timing of ovulation in an ubiquitous fashion amongst mammals (Herbison, 2020). In contrast, the sensitivity to testosterone negative feedback seems less stable in males amongst species and along life stages (Muller, 2017). For males, higher testosterone levels translate into better reproductive success, which makes the variation of testosterone plasma levels a selective force for many reproductive behaviours specific to males, as stated by the challenge hypothesis (Wingfield et al., 1990). Indeed, testosterone levels positively correlate with aggressiveness, territory defence, female fecundity, or even social instability of the alpha-male in a social group of primates (Muller, 2017). Inversely, in some primate species that exhibit paternal care and aggressive behaviour simultaneously, males can be insensitive to testosterone during the reproductive period (Marler et al., 2003; Trainor and Marler, 2001). For the grey mouse lemur specifically, males engage in sperm competition, and display all the anatomic and behavioural features associated with this trait: high testis size/ body size ratio, penile spines, aggressiveness and territorial defence behaviours (Aslam et al., 2002; Wedell et al., 2002). Hence the efficacy of the negative feedback of sexual steroids during the time of sexual activity seems to be lower in some highly territorial mammals, which is emphasized by our results in male mouse lemurs as they did not appear to lose sensitivity to testosterone and kept their reproductive activity contrary to females. However, although the lack of efficiency of deslorelin exposure in males in late winter might relate to specific life-history traits, the mechanism of testosterone desensitization of GnRH neuron cells, and its impact on LH and FSH remains to be demonstrated.

Although there is no intention relative to wildlife management here, one may question the relevance of testing the effect of deslorelin in captive animals. Actually, the favourable conditions of captivity have undoubtedly major impact on the general physiology of animals, especially on body composition (Hamalainen et al., 2014). However, the agenda for body mass fluctuations and for reproductive status are preserved in captivity (Perret and Aujard, 2001). This is partly due because seasonal animals lose sensitivity to a given photoperiod after some time, a phenomenon called photorefractoriness (Hozer and Perret, 2023). This is particularly critical in species that undergo periods of food scarcity and of low ambient temperatures, which have to be anticipated to ensure survival, especially by promoting fattening. This period coincides with the arrest of sexual activity and the complete shutdown of the reproductive axis, a feature that is also observed in captivity (Hozer and Perret, 2023). In summary, the maintenance of the alternation between long days and short days in captivity, though animals do not face food scarcity or cold, is sufficient to maintain the natural seasonal pattern of most physiological and behavioural components (Perret and Aujard, 2001; Terrien et al., 2017). This proves that the effects of the variations in daylength on the control of metabolism and reproduction are preserved under captive conditions, even in the absence of ecological constraints. This strongly suggests that the effect of deslorelin on the neuroendocrine regulation of the reproductive axis should be equivalent between captive and wild conditions.

Finally, deslorelin implants were put after 6 weeks of SD exposure, when all the animals, males or females, were sexually inactive – when the gonads do not have any endocrine nor exocrine activity (Perret and Aujard, 2001; Wingfield et al., 1990). However, we cannot rule out that an implantation of deslorelin later in the year, under LD photoperiod when the animals are sexually active but after the main period of maximal reproductive investment, may have been efficient to downregulate sexual activity in both males and females.

## Conclusion

Deslorelin implantation in late winter was efficient in downregulating reproductive activity in females grey mouse lemurs, but not in males. In an evolutionary perspective, a difference in response to a GnRH-agonist might originate from sex-specific life-history traits, notably related to the timing of reproductive effort. This study proposes to use deslorelin to evidence sex-specific regulatory pathways of sexual activity in fundamental studies. Moreover, males and females’ specific response to deslorelin could be better anticipated in a more applied context, especially to develop population control plans in seasonally breeding animals.
